# Serum 25-hydroxyvitamin D levels are associated with functional capacity but not with postural balance in osteoporotic postmenopausal women

**DOI:** 10.6061/clinics/2017(01)03

**Published:** 2017-01

**Authors:** Guilherme Carlos Brech, Emmanuel Gomes Ciolac, Mark D Peterson, Júlia Maria D’Andréa Greve

**Affiliations:** I Hospital das Clínicas da Faculdade de Medicina da Universidade de São Paulo, Instituto de Ortopedia e Traumatologia, Laboratório de Estudos do Movimento, São Paulo/SP, Brazil; II Universidade Ibirapuera, Fisioterapia, São Paulo/SP, Brazil; III Universidade Estadual Paulista Júlio de Mesquita Filho – UNESP, Faculdade de Ciências, Departamento de Educação Física, Bauru/SP, Brazil; IV University of Michigan-Medicine, Michigan, United States; V Faculdade de Medicina da Universidade de São Paulo, Instituto de Ortopedia e Traumatologia, São Paulo/SP, Brazil

**Keywords:** Elderly, Muscle Strength, Postural Balance, Sarcopenia, Timed Up and Go Test, Vitamin D

## Abstract

**OBJECTIVES:**

In post-menopausal women with osteoporosis, insufficient vitamin D levels decrease calcium fixation in the bones and calcium transport in the sarcoplasmic reticulum, which impairs muscle strength, possibly leading to detrimental consequences for the preservation of functional capacity and postural balance, fall prevention, and fracture risk. The aim of this study was to evaluate the association between vitamin D levels and knee muscle strength, postural balance and functional mobility among postmenopausal women with osteoporosis.

**METHODS:**

This cross-sectional study included 63 osteoporotic older women (aged 60.6±3.1 years). The subjects completed the Timed Up and Go Test to measure functional mobility, and postural balance was assessed on the AccuSway Plus portable force platform. Maximal strength was tested using an isokinetic dynamometer for knee flexion and extension. The subjects were assessed as a group and were divided into quartiles according to their vitamin D levels. Clinicaltrials.gov: NCT02771834.

**RESULTS:**

Vitamin D status was independently associated with the normalized peak torque of the knee extensors (β=0.59; *p*=0.04) and Timed Up and Go Test (β=-0.07; *p*<0.001). No between-group differences were observed in the demographic and clinical variables or postural balance; however, significant differences were observed in the Timed Up and Go Test, and the group with the highest vitamin D levels exhibited better performance than the group with the lowest vitamin D levels (*p*<0.001).

**CONCLUSION:**

The serum vitamin D levels were independently associated with normalized knee extension strength and functional mobility in postmenopausal women with osteoporosis.

## INTRODUCTION

Osteoporosis is a disease characterized by reductions in the bone mineral density (BMD) and deterioration of bone tissue, with increased fragility and risk of fractures. It is one of the largest public health problems, mainly due to the increasing age of the population 1. Osteoporosis affects approximately 55% of the population over the age of 50 years in the United States 2. In Brazil, the national health system (Sistema Único de Saúde - SUS) is also increasing its expenditures on treatments for fractures among older individuals, resulting in 20,778,000 hospital admissions in 2006 3. Women with osteoporosis often present a vitamin D deficiency, which further increases the risk of falls 4-6.

Optimal vitamin D levels are necessary for musculoskeletal health and function; however, over a billion people worldwide have a deficiency in their circulating vitamin D levels 5. Vitamin D is associated with muscular health 7 and contributes to the maintenance of muscle strength during ageing 5,8. Higher serum vitamin D concentrations have also been associated with greater muscle strength in healthy older individuals 4,5 who have suffered falls 9 and in those who have osteoporosis 6. However, these findings are currently being debated in the literature 10,11.

In postmenopausal women with osteoporosis, insufficient vitamin D levels reduce calcium fixation in the bones and calcium transport in the sarcoplasmic reticulum, which impairs muscle strength, possibly leading to detrimental consequences for the preservation of functional capacity and postural balance 5,6,12, fall prevention, and fracture risk 12. However, there is no consensus regarding the association of the serum vitamin D levels with muscle strength, functional capacity and postural balance in this specific population. Vitamin D supplementation improves muscle strength and postural balance but may have a limited influence on walking ability in older people without osteoporosis; however, subsequent studies are needed to examine these factors in postmenopausal women with osteoporosis 13.

Therefore, the aim of this study was to assess whether the serum vitamin D levels were associated with knee muscle strength, postural balance and functional mobility in postmenopausal women with osteoporosis.

## METHODS

### Study Location and Ethical Issues

The study was conducted at the Institute of Orthopedics and Traumatology, Hospital das Clínicas, School of Medicine, University of São Paulo, with approval granted by the Ethics Committee of the University of São Paulo (CaPPesq n° 320/09).

### Study Design

This cross-sectional study included 63 osteoporotic older women aged 55 to 65 years. The patients’ baseline characteristics are provided in Table 1. None of the subjects had pre-existing conditions related to the vestibular system, proprioceptive, auditory or neurological conditions, had not been prescribed any antipsychotic medication, had no restrictions on vigorous physical activity, had not been underwent surgery, and had not presented any injury to the legs in the previous six months. The subjects could have participated in physical exercises at a maximum of twice a week but not in strength exercises to remain eligible to participate in the study. According to the International Physical Activity Questionnaire (IPAQ), these subjects were considered to be irregularly active or active. Potential subjects were excluded from the study if they presented alterations in blood pressure or if they were not able to perform the test. The volunteers were analyzed together, and then divided into four groups, according to their serum vitamin D levels (Table 2). After the subjects had been provided an explanation of the study and signed the consent form, they were assessed in accordance with the evaluation protocol.

### Measurements

The following data were collected during the preliminary interviews and examinations: age, body mass, height, body mass index (BMI), ethnicity, race, age at the start of menopause, smoking status, and used of any hormonal replacement therapy (HRT) or calcium supplement. Moreover, dual-energy X-ray absorptiometry (DXA) values were recorded during each subject’s clinical examination.

All subjects were evaluated at the Laboratory of Kinesiology in the morning by the same professionals. The subjects were instructed not to perform any physical activity 24 hours prior to the tests, to wear comfortable and lightweight clothing, and to consume a low-calorie meal 2 hours before the visit.

The subjects then underwent the Timed Up and Go Test (TUGT) to measure functional mobility (time in seconds). The postural balance assessment (posturography) was performed on a portable force platform (AccuSway Plus, AMTI^®^, MA, USA). For data acquisition, the force platform was connected to a signal-amplifying interface box (PJB-101) that was linked to a computer using an RS-232 cable. The data were collected and stored using Balance Clinic^®^ software; the data were configured to a frequency of 100 Hz with a fourth-order Butterworth filter and a cutoff frequency of 10 Hz. All subjects performed the test with standardized positioning in relation to the maximum width of the support base (smaller than hip width), with the arms down, against the body and the head looking straight at a target. The base of support was drawn on a piece of paper located at a fixed position on the force platform and corresponded to the anatomical points of the distal hallux phalanx, fifth metatarsal head, and lateral and medial malleolus for each foot. Three 60-second measurements were collected when the subjects’ eyes were open (EO) and when they were closed (EC) . The arithmetic mean of the results was calculated from the three tests conducted under each condition and was processed using Balance Clinic^®^ software. The parameters used to measure the subjects’ stability when their eyes were open and closed were the mean square root of the displacement from the center of pressure (COP) in the mediolateral axis (XSD), the mean square root of the displacement from the COP in the anteroposterior axis (YSD), the mean velocity calculated from the total displacement of the COP in all directions (VAvg), and the total displacement area of the COP (Area95), as previously described 14,15.

Muscle force production was assessed using the Biodex^®^ Multi-joint System 3 (Biodex Medical^TM^, Shirley, NY, USA). The isokinetic dynamometer was calibrated thirty minutes prior to each test. After a standardized warm-up, the subjects were positioned for the concentric evaluation of extension and flexion movements of the knee joint. The subjects remained seated, with the hips at 90° of flexion and secured to the chair with belts. The test was started with the dominant limb. The limb was evaluated by aligning the lateral condyle of the femur with the mechanical axis of the dynamometer. All subjects performed four submaximal repetitions to familiarize themselves with the equipment, followed by a 60-second rest interval. Subsequently, two series of five maximal repetitions of knee extension and flexion were performed, starting with the dominant limb, with 60-second intervals between the series. Values from the second series were used to analyze the effects of motor learning on clinical isokinetic performance 16. Constant, standardized verbal encouragement was provided during the tests to promote maximum effort during contractions. The isokinetic variable was the maximum peak torque, which was corrected for body weight (PTQ/BW) and total work.

One milliliter of blood was collected from each subject and stored in a test tube to measure the serum 25(OH)D levels. The LIAISON^®^ 25 OH Vitamin D Total Assay kit (DiaSorin Inc., MN, USA) was used with the direct competitive chemiluminescent immunoassay for the quantitative determination of the total serum 25(OH) D levels.

### Statistical Analysis

All statistical analyses were performed using SigmaStat^TM^ 3.5 for Windows (Systat Software Inc., San Jose, CA, USA) and SAS software version 9.3 (SAS Institute, Cary, NC). The data are reported as the means and standard deviations (SD) or 95% confidence intervals (Cis). Multiple linear regression was used to evaluate the association between the vitamin D (25(OH)D) levels and all balance, strength and functional outcomes, after controlling for age. The normality of the residuals was tested using the Shapiro-Wilk test, and homogeneity of the variance of the residuals was tested with standard regression analyses. The data were also analyzed after the subjects were divided into quartiles according to their 25(OH)D levels. The Kolmogorov-Smirnov test was applied to ensure a Gaussian distribution of the data. One-way analysis of variance (ANOVA) was used to indicate differences in the variables that followed the Gaussian distribution among the groups, and Bonferroni’s post hoc analysis was used to identify significant differences between the groups, as indicated by the one-way ANOVA. The Kruskal-Wallis test (ANOVA on ranks) was used to indicate differences in the variables that did not follow a Gaussian distribution among the groups, and the Mann-Whitney post hoc test was used to identify significant differences between the groups, as indicated by the Kruskal-Wallis test. A minimum alpha level of *p*≤0.05 was used to determine statistical significance.

## RESULTS

The vitamin D levels ranged from 10.3 to 53.1 ng/mL (Table 1). Only 14 subjects (7.45%) were considered to have optimal vitamin D levels (>30 ng/mL), whereas 12.77% had insufficient levels (21-29 ng/mL) and 79.79% exhibited a deficiency (<20 ng/mL).

As shown in the multiple linear regression analysis, the vitamin D (25(OH)D) status significantly predicted a normalized peak torque of the knee extensors (β=0.59; *p*=0.04) and TUGT (β=-0.07; *p*<0.001) only after controlling for age. The demographics and clinical characteristics of the study population that had been divided into quartiles according to their 25(OH)D levels are presented in Table 2. No significant differences in the demographic and clinical variables were observed between the groups, with the exception of the 25(OH)D level, which showed significant differences between the groups.

No significant between-group differences were observed in the dominant and non-dominant leg PTQ/BW and total work (Table 3). Likewise, no significant between-group differences were observed in XSD, YSD, VAvg and Area95 in the postural balance assessment (Table 4).

**Figure 1:**
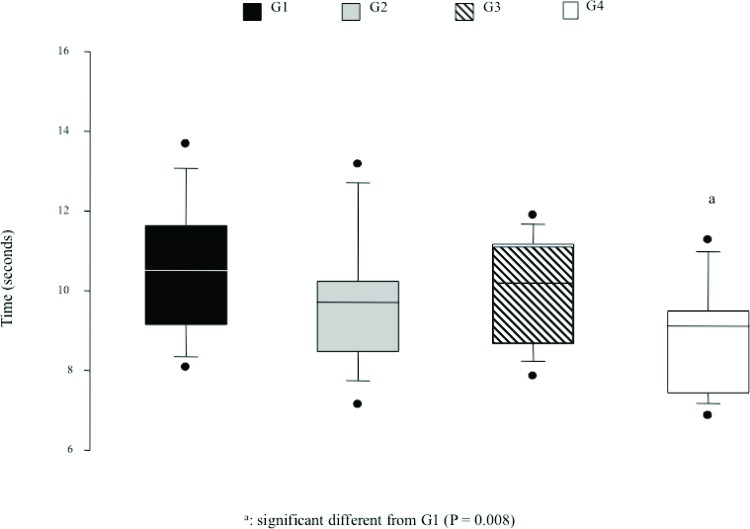
Timed Up and Go Test (TUGT) (95% CI).

However, significant differences were observed in the TUGT, consistent with the results of the multiple linear regression analysis. Women with higher 25(OH)D levels (G4) showed better performance than those with lower 25(OH)D levels (G1). The women in groups G2 and G3 showed intermediate TUGT performance that was not significantly different from either G1 or G4 (Figure 1).

## DISCUSSION

The principal finding of this study was that the serum 25(OH) vitamin D levels in postmenopausal women with osteoporosis were associated with knee extension strength and functional capacity. Vitamin D has been reported to influence neuromuscular transmission, cognition and extensor strength of the knee, important parameters for maintaining postural balance and functionality 6,17. Although the current findings did not support the hypothesis that the serum vitamin D levels contribute to balance, this study is one of the first to show an independent association between the serum vitamin D levels, strength and function within a sample of osteoporotic postmenopausal women.

Vitamin D supplementation increases the transverse section area of Type II fibers, improves proximal muscle strength and postural balance, and decreases the number of falls 8,18. Additionally, in as little as 3 months, vitamin D supplementation may increase muscle strength in older women with osteoporosis 19. Static and dynamic balance predict the risk of falls in older women, and the combination of vitamin D and calcium may reduce the risk of falls by as much as 22-29% 12,18. Even a short period of supplementation with vitamin D and calcium improves postural balance in older women, particularly in the anterior-posterior direction, and prevent falls and non-vertebral fractures 20. Vitamin D supplementation in older people prevented a single fall in a group of 15 people treated with vitamin D because vitamin D supplementation decreases the costs associated with falls 8 by preventing osteoporotic fractures and improving bone mineral density and muscle function 2.

In the present study, postural balance and vitamin D levels in osteoporotic women between the ages of 55 and 65 years were not associated, which has also been reported in another study 10. These results may have been influenced by the ages of the volunteers, even in a homogeneous sample regarding age. Some studies of patients who were older than those in our group found that postural instability is associated with low vitamin D concentrations 9 and the postural balance parameters can be improved with exercise intervention after three months 21. However, in another study, vitamin D supplementation did not improve physical function in older individuals 22.

Bischoff-Ferrari et al. 8 emphasized that high 25(OH)D concentrations (levels ≥40 ng/mL) are associated with better musculoskeletal function of the lower limbs and are recommended to maintain this functionality. However, in a cross-sectional study, impaired static balance was observed only in women with serum 25(OH)D levels of 25 nmol/L 23. An interventional study failed to show any significant improvement in the subjects’ static balance after vitamin D supplementation 24, and several studies failed to show any association between the 25(OH)D levels and quadricep strength 17,24. However, vitamin D supplementation at doses of 800 to 1,000 IU/d has a beneficial effect on balance and muscle strength 13; thus, the role of vitamin D as a contributing factor to functional capacity may well be mediated through the preservation of muscle function.

The association between vitamin D levels and performance on tests exploring mobility impairment has become a topic of much clinical discussion in the past two decades. The TUGT is a measure of functional mobility, muscle function, gait speed and balance 25. The concept of the TUGT is appealing because it describes a realistic mobility assessment that includes potential fall situations, such as getting into and out of a chair, walking, and turning around 25. Therefore, subjects must present muscle strength, particularly in the back and lower limbs, adequate extension and flexion of the hip and knee, postural control involving the interaction between the nervous and musculoskeletal systems to maintain balance, and cognitive skills to be able to complete the TUGT 26. Older subjects require more than 10 s to complete the TUGT and have an increasingly higher risk of falls 19.

The main components assessed by the TUGT are strength and functional capacity, which had important relationships with the 25(OH)D levels. These data corroborate the data reported by Toffanello et al. 27, who evaluated various functional tests, such as timed chair stands, gait speed, and a 6-minute walking distance test. Of all the performance measurements considered, the 6-minute walking distance test was the motor test that was most closely related to the vitamin D status in both sexes.

Vitamin D may indirectly contribute to the TUGT by regulating the musculoskeletal system, particularly muscle strength and neuromuscular coordination. In a study involving forty-two older women, the serum vitamin D concentrations were not significantly correlated with basic functional mobility, and higher serum vitamin D concentrations are not associated with a shorter time needed to complete the TUGT 26. However, in a recent study of 540 elderly women, TUGT performance was associated with age, exercise type and the serum 25(OH)D levels 28.

Although the causal effects of vitamin D on postural balance, muscle strength, and functional capacity are controversial, various studies have indicated an improvement in these parameters when subjects are provided vitamin D supplements, particularly in older individuals. Schacht and Ringe 19 conclude that daily treatment with 1 mcg of alfacalcidol is safe, increases muscle power, muscle function, and walking distance, decreases the fear of falls, and improves the performance in the TUGT and fall risk test. Even a single mega dose of vitamin D may be sufficient to increase the quality of life, decrease non-specific musculoskeletal pain, and prevent falls by improving functional mobility in older individuals 29.

### Limitations

The literature on the influence of the vitamin D levels on muscle function, postural balance, gait, and functional independence is inconsistent. Regarding the tests conducted, the TUGT demands more action of the systems required for balance control. Therefore, vitamin D deficiency might accelerate and aggravate the process of functional loss that occurs during ageing and vitamin D replacement is an important adjuvant factor.

Similar to all cross-sectional investigations, a limitation of this study is its inability to unravel the cause-effect relationship between exposure and the outcomes. Indeed, studies determining whether low vitamin D levels “cause” lower knee extension strength and decrease functional mobility, or whether secondary conditions, such as general frailty syndrome, are drivers of poor nutrition habits, are interesting and complex. Nevertheless, based on these results, vitamin D levels are associated with knee extension strength and improved performance on the TUGT (dynamic equilibrium) in a group of postmenopausal women with osteoporosis. Another potential limitation is that we did not determine the vitamin D intake via diet or supplementation; therefore, we cannot determine whether differences in the vitamin D levels were due to exposure to the sun or oral intake. We did not measure other serum factors related to vitamin D metabolism, including calcium, parathyroid hormone, phosphorus, and sex hormone levels; thus we cannot determine whether these factors contributed to the “vitamin D effect.”

### Clinical implications

A healthy ‘musculoskeletal system’ is required for individuals to maintain independence as they age. Guided by the muscle, bones continuously adapt to their function. The deterioration of muscle power, function and balance contributes to the increased risk of falls and related fractures in older subjects with osteoporosis. Thus, obtaining a better understanding of the importance of vitamin D in the musculoskeletal system and of its role in maintaining muscle strength, postural balance and functional capacity, is essential to plan preventive strategies to prevent falls in women with osteoporosis.

## AUTHOR CONTRIBUTIONS

Brech GC was responsible for data collection, data analysis and preparation of the manuscript. Ciolac EG was responsible for data analysis and preparation of the manuscript. Peterson MD edited the manuscript. D’Andrea Greve JM supervised the study and was responsible for manuscript editing.

## Figures and Tables

**Table 1:** Baseline characteristics of the study participants.

Variable	Mean (SD)
Age (years)	60.6 (±3.1)
25(OH) D (ng/mL)	24.2 (±9.2)
Body weight (kg)	59.8 (±10.5)
Height (m)	1.52 (±0.1)
Body mass index (kg/m^2^)	25.8 (±4.2)
SD	- 3.01 (±0.5)
Femoral neck	
Bone mineral density (g/cm^2^)	0.70 (±0.2)
SD	- 1.81 (±0.7)
Total hip	
Bone mineral density (g/cm^2^)	0.78 (±0.1)
SD	- 1.63 (±0.8)
Physical activity (IPAQ)	
Active	44%
Irregular activity	56%
Menopause	
Age when menopause started (years)	46.8 (±5.0)

25(OH) D: 25-hydroxyvitamin D; SD: standard deviation.

**Table 2:** Demographic and clinical characteristics of the study participants in each group (G).

Variable	G1 (n=15)	G2 (n=16)	G3 (n=16)	G4 (n=16)
25(OH)D (ng/mL)	14.0±3.3	19.5±2.3^a^	24.0±2.3^b^	36.7±7.7^c^
Age (years)	61.7±3.6	60.8±2.9	58.8±2.9	61.3±2.8
Body mass index (kg/m^2^)	25.1±4.6	26.2±3.5	25.6±3.7	26.2±5.2
Ethnicity				
White	10 (66.7%)	14 (87.5%)	15 (93.7%)	11 (68.7%)
Black	4 (26.7%)	2 (12.5%)	1 (6.3%)	4 (25%)
Other	1 (6.6%)	0 (0%)	0 (0%)	1 (6.3%)
Menopause (years)*	16.2±7.3	14.0±5.8	13.1±5.2	12.7±3.9
Smoking	2 (13.3%)	3 (18.7%)	1 (6.2%)	3 (18.7%)
HRT	6 (40%)	11 (68.7%)	9 (56.2%)	10 (66.7%)
Calcium supplementation	8 (53.3%)	8 (50%)	15 (93.7%)	6 (37.5%)
Bone mineral density (g/cm^2^)				
Vertebral column	0.72±0.07	0.75±0.05	0.74±0.06	0.72±0.09
Femur	0.77±0.06	0.76±0.09	0.76±0.07	0.81±0.12
Femoral neck	0.69±0.06	0.71±0.08	0.70±0.05	0.76±0.09

25(OH) D: 25-hydroxyvitamin D; HRT: hormonal replacement therapy.

* Time since the start of menopause.

a different from G1 (P < 0.001)

b different from G1 and G2 (P < 0.001)

c different from all groups (P < 0.001).

**Table 3:** Isokinetic performance of the study participants in each group (G).

Variable	G1 (n=15)	G2 (n=16)	G3 (n= 6)	G4 (n=16)
Knee extension				
PTQ/BW dominant leg (%)	150.3±27.8	152.8±27.9	148.2±34.4	158.8±29.5
PTQ/BW non-dominant leg (%)	146.5±30.0	152.9±20.2	149.4±24.5	152.4±29.8
Total work dominant leg (J)	279.0±52.2	297.3±89.4	268.0±61.1	298.0±61.3
Total work non-dominant leg (J)	277.7±47.5	320.8±66.4	272.5±49.8	290.9±61.2
Knee flexion				
PTQ/BW dominant leg (%)	71.3±23.2	77.3±20.7	74.7±13.3	83.6±24.7
PTQ/BW non-dominant leg (%)	73.3±21.3	73.5±15.9	73.2±16.6	76.6±15.8
Total work dominant leg (J)	148.1±40.0	165.9±54.8	145.3±37.4	168.4±47.1
Total work non-dominant leg (J)	146.3±36.0	166.3±50.0	146.1±32.2	156.8±39.0

XSD: mean square root of the displacement from the COP in the mediolateral axis; YSD: mean square root of the displacement from the COP in the anteroposterior axis; VAvg: mean velocity calculated from the total displacement of the COP in all directions; Area95: total displacement area of the COP.

**Table 4:** Balance performance of the study participants in each group (G).

Variable	G1 (n=15)	G2 (n=16)	G3 (n=16)	G4 (n=16)
**Eyes open**				
XSD (cm)	0.27±0.09	0.21±0.06	0.25±0.08	0.23±0.06
YSD (cm)	0.37±0.08	0.39±0.11	0.39±0.14	0.38±0.10
VAvg (cm/sec)	0.79±0.20	0.75±0.13	0.77±0.18	0.76±0.12
Area95 (cm^2^)	1.78±0.87	1.46±0.68	1.77±1.00	1.48±0.50
**Eyes closed**				
XSD (cm)	0.27±0.11	0.22±0.07	0.29±0.15	0.22±0.06
YSD (cm)	0.38±0.12	0.41±0.10	0.45±0.15	0.41±0.16
VAvg (cm/sec)	1.10±0.31	0.98±0.23	1.10±0.37	1.03±0.20
Area95 (cm^2^)	20.3±1.28	1.63±0.70	2.49±1.64	1.71±0.86

XSD: mean square root of the displacement from the COP in the mediolateral axis; YSD: mean square root of the displacement from the COP in the anteroposterior axis; VAvg: mean velocity calculated from the total displacement of the COP in all directions; Area95: total displacement area of the COP.
